# Genes expression profiling of alveolar macrophages exposed to non-functionalized, anionic and cationic multi-walled carbon nanotubes shows three different mechanisms of toxicity

**DOI:** 10.1186/s12951-020-0587-7

**Published:** 2020-02-24

**Authors:** Sara Nahle, Hilary Cassidy, Mélanie M. Leroux, Reuben Mercier, Jaafar Ghanbaja, Zahra Doumandji, David Matallanas, Bertrand H. Rihn, Olivier Joubert, Luc Ferrari

**Affiliations:** 1 Nanomaterials and Health, Team 403, Institute Jean Lamour UMR 7198 du CNRS, Université de Lorraine, 54000 Nancy, France; 2 Systems Biology Ireland, School of Medicine, University College Dublin, Belfield, Dublin 4, Ireland

## Abstract

Functionalized multi-walled carbon nanotubes (MWCNT) have become the focus of increased research interest, particularly in their application as tools in different areas, such as the biomedical field. Despite the benefits associated with functionalization of MWCNT, particularly in overcoming issues relating to solubility, several studies have demonstrated that these functionalized nanoparticles display different toxicity profiles. For this study, we aim to compare NR8383 cells responses to three well-characterized MWCNT with varying functional groups. This study employed cytotoxicity assays, transcriptomics and proteomics to assess their toxicity using NR8383 rat alveolar macrophages as an in vitro model. The study findings indicated that all MWCNT altered ribosomal protein translation, cytoskeleton arrangement and induced pro-inflammatory response. Only functionalized MWCNT alter mTOR signaling pathway in conjunction with increased *Lamtor* gene expression. Furthermore, the type of functionalization was also important, with cationic MWCNT activating the transcription factor EB and inducing autophagy while the anionic MWCNT altering eukaryotic translation initiation factor 4 (EIF4) and phosphoprotein 70 ribosomal protein S6 kinase (p70S6K) signaling pathway as well as upregulation *Tlr2* gene expression. This study proposes that MWCNT toxicity mechanisms are functionalization dependent and provides evidence that inflammatory response is a key event of carbon nanotubes toxicity.

## Introduction

Carbon nanotubes (CNT) are increasingly used in different sectors including the biomedical one due to their distinctive properties. We have monolayer CNT, called single walled carbon nanotubes (SWCNT) and multilayer CNT, called multi walled carbon nanotubes (MWCNT). In the last few years, 736 metric tons of these CNT, were used for energy and environmental applications, with these figures constantly increasing over time [1]. The global market of CNT is estimated to grow from USD 4.55 billion in 2018 to an estimated USD 9.84 billion by 2023 [2]. Thus, exposure to CNT has become a major environmental issue and a potential human health risk. The main route by which CNT enter the environment is through biomechanical degradation or combustion of nanocomposites-based products leading to air contamination [3, 4]. Additionally, CNT can be used in water treatment to remove organic, inorganic and biological pollutants from water which could potentially pollute aquatic ecosystems [5]. Furthermore, through irrigation CNT can enter land to soil leading to plants contamination and subsequent incorporation into food chain and accumulation in water waste [6]. Currently, protective measures taken in response to potential CNT exposure are limited to specific equipment for workers with little progress reported on an environmental scale.

Recently, several studies have focused on carbon nanotubes functionalization. It has been demonstrated that carboxyl and amino groups can be added endowing the CNT with new characteristics and subsequently making them suitable for more applications [7]. Carboxylic functionalization increases the solubility of CNT in comparison to non-functionalized pristine CNT thus aiding in developing nanocomposites of high quality and distinctive optical properties [8]. Moreover, it enhances the CNT electrical conductivity and thermal stability to obtain, for example, a better conductive cotton textile [9]. Carboxylated CNT are suggested to be used in different products ranging from biomedical and therapeutic applications, gene delivery, cancer diagnosis to vaccination [10–12]. Likewise, amino-functional groups anchored to CNT also enable numerous applications because of their specific chemical characteristics such as high electron donation capacities and enhanced nucleophilicity. These amino-CNT are used for biosensor fabrication, electrocatalysts and nanocomposites preparation, and metal ions absorption [13]. Additionally, it has been shown that the attachment of amino-functional groups to CNT can promote CNT uptake by cells, which has led to the potential use of functionalized CNT as drugs carriers.

Given the variety of applications that functionalized CNT possess, studies have been developed to understand the impact of this functionalization on their toxicity. Zhou et al. [14] demonstrated that COOH functionalizing reduced CNT cytotoxicity in human lung cancer cells (A549), in comparison to the pristine form, proposing that carboxyl group increased their degree of aggregation. This decrease in CNT toxicity following carboxyl functionalization has been confirmed by several studies performed in A549 human lung cancer cells [17] and RAW264.7 macrophages [18, 19]. Studies have hypothesized that this decrease can be related to the increase of NPs aggregation [17, 18], or to the increase of CNT biodegradability [19]. Furthermore, Jang et al. [15] demonstrated that carboxylate CNT trap toxic lead ions which reduce their cytotoxicity induced in *Daphnia magna*. However, it is important to note that there are contradictory reports, with a study by Cinzia et al. showing that carboxyl functionalization induced a toxic response in BEAS-2B cells, with increased inflammatory response and cytotoxicity in comparison to pristine CNT exposure. This finding is supported by other studies showing that carboxylic functionalization enhanced CNT cytotoxicity in HUVEC [16] and H596 cells [17, 18], and increased DNA damage resulting in apoptosis and cell death in MCF-7 cell line [19, 20]. Importantly, the possible relevance of CNT amino functionalizing is characterized worse. To date, it has been reported that there is no difference in cytotoxicity induced in fibroblasts between amino functionalized and pristine CNT [21]. There might even be a mitigation of the toxicity induced by pristine CNT Raw264.7 macrophages and PC12 cells [22]. Given the different endpoints which seems to be particular to each cell type and suggesting that macrophages could be more sensitive than other cells like epithelial ones, it would be interesting to focus on how functionalizing is critical for phagocytic cells. It is clear that functionalization could be one of the most relevant determinants of CNT toxicity and reinforces the urgent need for further investigations into the toxic effect of these functionalized CNT. Therefore, comparative analysis was carried out in this study to understand the toxicity mechanisms caused by differential functionalization among three MWCNT: NM403 (non-functionalized pristine), NRCWE-042 (anionic) and NRCWE-049 (cationic). Most of inhaled nanoparticles are trapped in the pulmonary alveolar region of rats, and they are principally in contact with macrophages, the first and primary cell types that process nanoparticles, mediating host inflammatory and immunological biological responses [23, 24]. Therefore, it was interesting the use of NR8383 rat alveolar macrophage cell line for this study. These cells have been commonly used to predict CNT effects in the respiratory tract [25, 26] but to our knowledge, it is the first use aiming to study CNT functionalization effects. In addition, taking into consideration the similarity of rat and human genome, these data focusing on genes expression variation due to carbon nanotubes exposure, can be concluding for humans.

## Experimental

### Cell culture

NR8383 rat alveolar macrophages were purchased from American Type Culture Collection (ATCC^®^ CRL2192™; Manassas; USA). Cells were cultured in Dulbecco’s Modified Eagle Medium DMEM, high glucose [27, 28] (Sigma-Aldrish; St. Louis; USA), supplemented with 15% Fetal Bovine Serum, FBS (Sigma-Aldrish; St. Louis; USA), 100 U/mL penicillin and 100 g/mL streptomycin, 4 mM l-glutamine and 0.25 μg/mL of amphotericin B (Sigma-Aldrish; St. Louis; USA). Cells were cultured at 37 °C in humidified chamber with 5% CO_2_. N8383 cells were passaged every 3 days.

### Nanomaterial dispersion and characterization

#### Nanomaterials

NM403 were obtained from Joint Research Centre, JRC, (Italy) while NRCWE-042 and NRCWE-049 were obtained from the National Research Centre for the Working Environment, NRCWE, (Denmark) via SmartNanoTox project partners. The physical and chemical properties and purity percentage of these MWCNT are indicated in Table 1. The MWCNT were suspended in DMEM medium supplemented with 2% FBS at a concentration of 2 mg/mL and are sonicated with probe sonicator cooled on ice water (Philip Harris Scientific; Lichfield; UK) using different dispersion settings as it shown in Table 1. Preliminary experiments showed that the stability of the preparation was up to 48 h. All experiments were realized immediately after sonication to ensure a good dispersion of the MWCNT.

**Table 1:** Dispersion conditions and MWCNT properties provided by supplier (a) and measured using DLS (b), in addition to purity retrieved from literature (c) [29]

Name	MWCNT type	Length^a^ (nm)	Diameter^a^ (nm)	Specific surface^a^ (cm^2^/cm)^2^	Probe (mm)	Amplitude	Time (min)	Z-average^b^ (nm)	Zeta potential	Pdi^b^	Purity (%)^c^
NM403	Pristine	400	12	135	11	10	15	217.2 ± 70.3	− 13.005 ± 0.025	0.383 ± 0.013	> 90
NRCWE-042	Functionalized –COOH	723	21	141	5	30	15	210.5 ± 45.2	− 28.125 ± 0.307	0.279 ± 0.019	> 95
NRCWE-049	Functionalized –NH_2_	731	14	199	5	30	20	210.4 ± 81.3	+ 7.021 ± 0.563	0.466 ± 0.025	> 99

#### Particle size and zeta potential

The measurements of hydrodynamic diameter average, polydispersity index, PDI, and zeta potential of the nanomaterials with the principle of dynamic light scattering, DLS, were performed with Malvern Nano Zetasizer (Malvern Inc.; Worcs; UK). MWCNT from stock solution (2 mg/mL) were diluted in medium 0% FBS to a concentration equal to 200 µg/mL retained for DLS measurement.

#### TEM characterization

For transmission electron microscopy, TEM, 50 µL of CNT (NRCWE-042 and NRCWE-049) suspension was deposited onto a carbon-coated copper grid. After drying, the sample was negatively stained by uranyl acetate (3%) in deionized water. Preparations were observed using a CM12 Microscope (Philips; Amsterdam; The Netherlands) operated at 80 kV. CNT diameter was determined by observing 114 particles for each MWCNT. Representative TEM images for NM403 have been shown in a previous study [30].

### Cytotoxicity assay

#### Viability test (WST-1)

WST-1 assay was performed as previously described [30]. Briefly, NR8383 cells were seeded in 96 well-plate with 5 × 10^3^ cells/100 µL/well and were grown at 37 °C under a 5% CO_2_ atmosphere in humidified incubator overnight. The following day the plate was centrifuged (800×*g*, 10 min) and the medium 15% FBS was removed and replaced with another one with 0% FBS to which CNT at different concentrations prepared from the stock solution was added so that the final concentrations indicated were obtained: 6.25, 12.5, 25, 50, 100 and 200 μg/mL that correspond respectively to 2.5, 5, 10, 20, 40, 80 cm^2^/cm^2^ (NP surface/Cell surface) for NM403; 3, 5.5, 11, 21.5, 43, 86 cm^2^/cm^2^ for NRCWE-042 and 4, 7.5, 15, 30, 60, 120 cm^2^/cm^2^ for NRCWE-049 [14, 31]. These exposures were performed in DMEM media without FBS. The cells were then incubated with 5 μL of WST-1 Cell Proliferation Reagent (Roche; Boulogne; France) for 2 h at 37 °C, 5% CO_2_. The absorbance was measured using iMarK™ Microplate Reader (BIO-RAD Laboratories; Osaka; Japan) at 450 nm. Based on WST-1 results, the inhibitory concentration, IC_50_, was calculated according to Reed and Muench formula [32].

#### Lactate dehydrogenase (LDH) cytotoxicity assay

The assay was performed using the LDH Cytotoxicity Detection Kit (Roche; Boulogne; France). Cells were seeded in 96 well-plate with 5000 cells/100 µL/well and treated in the same manner as described for the viability assays. The assay was conducted following the manufacturer instructions. A lysis solution of 10% triton serves as positive control. LDH activity in the supernatant was quantified using an iMarK™ Microplate Reader (BIO-RAD Laboratories; Osaka; Japan) at 490 nm wavelength and 630 nm as reference wavelength. Medium and lysates were used as negative and positive control respectively.

### RNA extraction

RNA extraction was performed as previously described [33]. Briefly, following NR8383 cells exposure for 4 h to ¼ IC_50_ of each MWCNT (0.8 cm^2^/cm^2^ for NM403, 6.8 cm^2^/cm^2^ for NRCWE-042 and 1.2 cm^2^/cm^2^ for NRWE-049), supernatants were collected for cytokine array assay and the membranes were disrupt by adding 1 mL of Trizol Extraction Reagent (OMEGA Bio‐Tek; Guang zhou; China), followed by the addition of 200 µL of chloroform (Carlo Erba reagents; Normandie; France). Samples were centrifuged at 800*g* for 15 min. Then, 500 µL of Isopropanol (Carlo Erba reagents; Normandie; France) were added to 350 µL of supernatant. The precipitates were subjected to 2 washing steps with 80% ethanol and incubated for 10 min at 60 °C to remove ethanol, followed by dissolution in 35 µL RNase-free water. All RNA samples were of high purity and integrity, as demonstrated by A260/A280 ratios greater than 2 as displayed by BioSpec-nano Spectrophotometer (SHIMADZU; Kyoto; Japan), RNA integrity numbers were above 9.0 checked by RNA 6000 Nano Reagents Kit using Bioanalyzer™ 2100 (Agilent Technologies; Waldbron; Germany).

### Microarray hybridization

Microarray were prepared as previously described [34]. Briefly, 100 ng of RNA from each sample was labeled with cyanine 3-CTP using Low Input Quick Amp Labeling assay™ (Agilent Technologies; Waldbron; Germany) according to the manufacturer’s protocol. Labeled cRNAs were purified and hybridized onto Agilent G4853A SurePrint G3 Rat version 3 GE 8*60 K microarrays (Agilent Technologies; Waldbron; Germany) allowing a full coverage of the rat transcriptome. Microarray slides were scanned on an Agilent G2505C microarray scanner™ with a 3 μm resolution.

### Pathway analysis of microarray data

#### Gene expression omnibus (GEO) database

Raw intensity data were extracted using Agilent Feature Extraction Software version 11.0. Experiments were performed according to MIAME standards [35]. This data have been uploaded to the NCBI Gene Expression Omnibus database [36].

#### GeneSpring

Raw data were first normalized using GeneSpring GX 13.0 software (Agilent Technologies, UK) with Lowess’ method. After, a principal component analysis (PCA), was done using GeneSpring as a quality control step where the outlier’s samples were removed. In order to identify genes whose expression level was significantly modified, Student’s t-test followed by Benjamini–Hochberg False Discovery Rate correction were used and filtering criteria were then applied. Genes for which fold-changes (FC) for exposed vs. matched controls was at least 1.5 in either direction, and with p-values < 0.00 were considered significantly differentially expressed and were used in the following analysis.

#### Ingenuity pathway analysis (IPA)

The data was analyzed by Ingenuity Pathway Analysis (IPA) (Ingenuity Systems, www.ingenuity.com), which predicts canonical pathways which are changing based on gene expression (FC > 1.5; FC > 3; FC > 6), as well as identifying upstream regulators which drive changes in gene expression. Finally, IPA can identify diseases or functions related to these gene changes. Venn diagrams were created comparing the significant genes across the three different treatment groups (NM403, NRCWE-042 and NRCWE-049). The data was also analyzed using DAVID Functional Annotation Bioinformatics Microarray analysis (https://david.ncifcrf.gov/) and String (https://string-db.org/), which creates functional protein association networks.

### Proteomic analysis

In order to know if genes expression variations due to CNT exposure will be extrapolated also at the protein level, thus modifying cell final phenotype, a proteomic analyses was performed. Principally, proteomic data will be used to validate certain transcriptomic endpoints (such persistence of inflammatory response or DNA damage) after 24 h taking onto consideration the duration of translation process.

#### Single-pot solid-phase-enhanced sample preparation (SP3) for whole cell proteomics

The so called SP3 method was employed to analyze the NR8383 macrophage cells global proteome [37]. Both hydrophobic and hydrophilic Sera-Mag Speed Bead Magnetic carboxylate modified particles were employed in a 1:1 (v/v), (GE Healthcare; Illinois; USA), preparing the beads for use by rinsing them in MS water (Fisher Scientific, Cat # 10777404) twice before final reconstitution in a volume of MS water equal to the starting volume [37]. The reconstituted beads can be stored at 4 °C until required. The SP3 digest was performed according to the protocol of Hughes. In brief, cell pellets were resuspended in 100 μL lysis buffer:6 M urea, 2 M thiourea, 50 mM MOPS. The lysates were reduced and alkylated in 5 μL 0.2 M 1,4-dithiothreitol (DTT) (Sigma AldrichSt. Louis; USA) and 5 μL of 0.4 M iodoacetamide (IAA) (Sigma Aldrich, St. Louis; USA), respectively. After reduction and alkylation, 100% acetonitrile (Sigma Aldrich, St. Louis; USA) was added to each sample to a final concentration of 70% acetonitrile. Next, 10 μL of the prepared bead mix was added to the lysate and samples were rotated at room temperature for 18 min. Subsequently, beads were immobilized by incubation on a DynaMag-2™ stand (Thermo Fisher, Oslo; Norway) for 2 min. The supernatant was discarded, and the beads were washed with 70% (v/v) ethanol, and 100% acetonitrile. Beads were resuspended in 50 μL of 50 mM ammonium bicarbonate (NH_4_HCO_3_) (Sigma Aldrich, CAT # 09830-500G), supplemented with sequence grade trypsin (Promega; Madison WI; USA) at an enzyme-to-protein ration of 1:25 (w/v). After overnight hydrolysis at 37 °C, the beads were vortexed gently and an additional 8 μL of the prepared bead slurry was added to each sample and rotated for 18 min atRT. The beads were immobilized by incubation on a DynaMag-2™ stand, washed once with 50 mM NH_4_HCO_3_ and 100% acetonitrile. Peptides bound to the beads were eluted using MS grade water with intermittent vortexing for 5 min, centrifuged at 15,000 rcf at 4 °C for 15 min. The beads were immobilized and the supernatant containing purified peptides was transferred into MS vials and acidified by adding 2 µL of acetic acid. Samples were stored at 4 °C until analysis by mass spectrometry.

#### Filter aided sample preparation (FASP) of supernatants for proteomics

Supernatants were obtained from experiments exposing NR8383 cells to the three different MWCNT. Protein content was determined using a NanoDrop 2000 spectrophotometer (Thermo Scientific; Waltham, USA). Filter-aided sample preparation, FASP, was performed as previously described [38]. Briefly, 50 μg protein was reduced by adding IM DTT to a final concentration of 0.1 M DTT. Samples were mixed with 8 M urea in 0.1 M Tris–HCl, pH 8.9 (UA buffer) and loaded onto ultracentrifugation units of nominal molecular weight cutoff 10.000 (Sartorius Stedim Biotech; Gottingen; Germany). Traces of detergents were removed by washing the samples twice with UA buffer. Proteins were alkylated with iodoacetamide prepared in UA buffer and the samples were incubated in the dark at room temperature for 20 min. The filter units were washed twice with 50 mM, NH_4_HCO_3_, and the filter units were transferred to fresh collection tubes. Proteins were hydrolysed overnight at 37 °C using sequence grade trypsin (Promega, Madison WI, USA) at an enzyme-to-protein ratio of 1:50 (w/w). Peptides were recovered by centrifuging the filter units, then washing the filters once with 50 mM NH_4_HCO_3_ and repeating the centrifugation, combining the flow-throughs. The samples were measured on a NanoDrop 2000 spectrophotometer™ to calculate the protein concentrations. Twenty micrograms of the tryptic digests were loaded separately and desalted on C18 Stage tip as described by Rappsilber [39]. Following elution of the peptides from the Stagetip the samples were lyophilized in a CentriVap Concentrator with open caps for approximately 10–15 min, until approximately 5 μL volume remained. The remaining sample was resuspending in trifluoroacetic acid, TFA, solution. The sample was placed in mass spectrometry vials and stored at 4 °C until analysis by mass spectrometry.

#### Mass spectrometry settings

Each treatment was run with four biological replicates which were then run with two technical replicates on a Thermo Scientific Q Exactive mass spectrometer connected to a Dionex Ultimate 3000 (RSLCnano™) chromatography system. Each sample was loaded onto a fused silica emitter (75 μm ID), pulled using a laser puller, Sutter Instruments P2000™ (Novato; CA; USA), packed with Reprocil Pur™ (Dr. Maisch, Ammerbuch-Entringen, Germany), C18 (1.9 μm; 12 cm in length) reverse phase media and were separated by an increasing acetonitrile gradient over 90 min at a flow rate of 250 nL/min direct into a Q-Exactive MS. The MS was operated in positive ion mode with a capillary temperature of 320 °C, and with a potential of 2300 V applied to the frit. All data was acquired while operating in automatic data dependent switching mode. A high resolution (70,000) MS scan (300–1600 m/z) was performed using the Q Exactive™ to select the 12 most intense ions prior to MS/MS analysis using high-energy collision dissociation, HCD.

#### Maxquant analysis of mass spectrometry data

Proteins were identified and quantified by MaxLFQ [Cox 2014] by searching with the MaxQuant version 1.5. Modifications included C carbamylation (fixed) and M oxidation (variable). The resulting data was then analysed using online platforms such as String and PANTHER.

### Cytokine array

In order to verify the induction of inflammation by these MWCNT, at a protein level, a cytokine array was prepared. Supernatants from exposed cells stored at − 80 °C were analysed using Proteome Profiler Rat XL Cytokine array™ (R&D Systems Europe, Abingdon, UK) according to manufacturer’s instructions. The supernatants corresponding to cells exposed to the ¼ IC_50_ of each MWCNT as well as a negative control (unexposed cells) were analysed. This corresponds to 0.8 cm^2^/cm^2^ for NM403, 6.8 cm^2^/cm^2^ for NRCWE-042 and 1.2 cm^2^/cm^2^ for NRCWE-049. Briefly, after blocking the membrane, supernatants were incubated with the membrane for 2 h at room temperature under smooth agitation. After washing, the membrane was incubated with a mixture of diluted biotinylated antibodies for 1 h at room temperature under smooth agitation. After washing, the membrane was incubated with a solution of diluted streptavidin-horseradish Peroxydase for 30 min at room temperature under smooth agitation. Finally, after washing, a mixture of H_2_O_2_ and luminol was added for chemiluminescence reaction. Revelation was performed on a ChemiDoc Touch™ (BioRad, Strasbourg, France). Densitometric analysis was performed on ImageLab™ (BioRad, Strasbourg, France).

#### Statistical analysis

Four biological replicates are used for each experiment. Statistical differences were determined by one-way analysis of variance (ANOVA) followed by Tukey–Kramer test (Dunnett’s test) for cell viability.

## Results

### Nanoparticle characterization

**Fig. 1:**
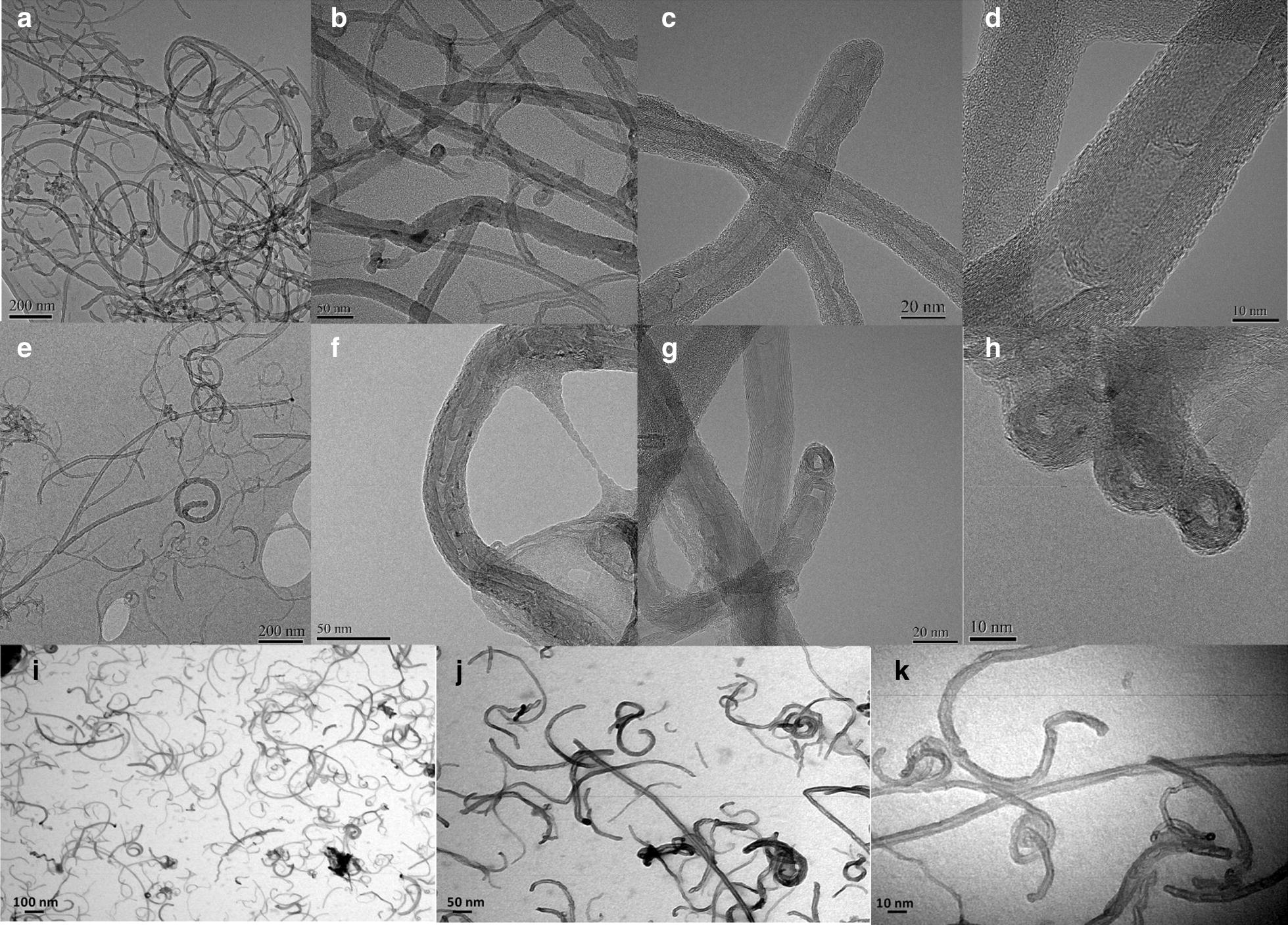
Transmission electron microscopy for NRCWE-042 (**a**–**d**), NRCWE-049 (**e**–**h**) and NM403 (**i**, **k** and **l**)

MWCNT mean hydrodynamic diameters, zeta potential and polydispersity index, determined by DLS were shown in Table 1. All MWCNT studied in the media with 2% FBS displayed similar Z-average. The length calculated by TEM was 750 ± 150 nm for NRCWE-042 and 600 ± 100 nm for NRCWE-049 and 300 ± 90 nm for NM403 [30] (Fig. 1).

### Cell viability

All The IC_50_ of NM403, the non-functionalized MWCNT, and the amino-group functionalized CNT NRCWE-049 were found to be similar, 3.2 cm^2^/cm^2^ and 4.8 cm^2^/cm^2^ respectively. However, the IC_50_ for the carboxylated CNT (NRCWE-042) was shown to be much higher at 27.2 cm^2^/cm^2^. The IC_50_ determined in this assay were utilized to the doses to be employed in the transcriptomics study, which was set at ¼ IC_50_.

**Fig. 2:**
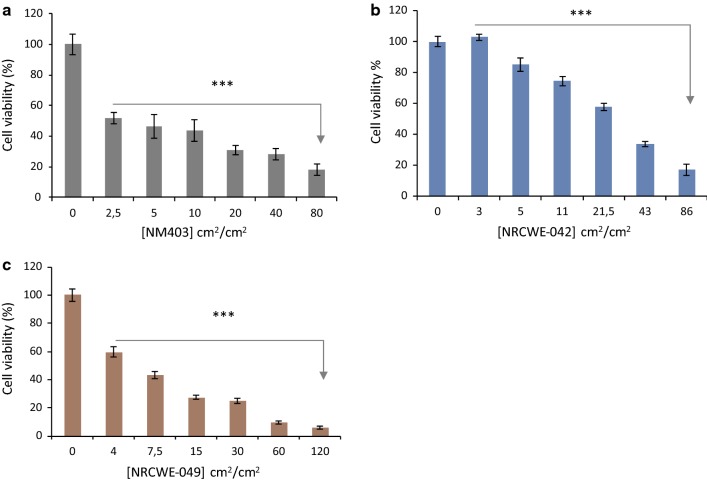
Cytotoxicity of MWCNT on NR8383 cells. The toxicity was evaluated with WST-1 test. NR8383 cells were exposed to NM403 range from 0 to 80 cm^2^/cm^2^ (**a**), to NRCWE-042 range from 0 to 86 cm^2^/cm^2^ (**b**) and to NRVWE-049, range from 0 to 120 cm^2^/cm^2^ (**c**). Data represents the mean ± SD of four independent experiments. ***p < 0.001 vs non-treated cells. ANOVA followed by Dunnett’s multiple comparison test

Assessment of cytotoxicity by means of the LDH assay showed that only NM403 induced loss of NR8383 cell membrane integrity following a 24 h exposure period. A significant increase of 12.5% and 20% of LDH release was evidenced respectively at 10 and 20 cm^2^/cm^2^ doses (data not shown; Fig. 2).

### Transcriptomic study

**Fig. 3:**
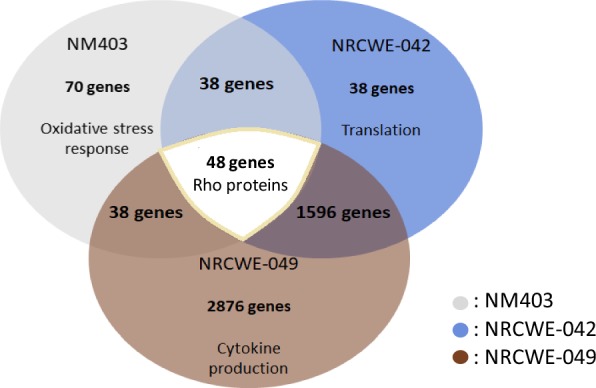
Venn diagrams based on transcriptomic analysis results showing the number of common genes dysregulated [45] after NR8383 cells exposure to three MWCNT and the number of genes specific to each exposure at fold change, FC ≥ 1.5 (70 for NM403, 38 for NRCWE-042 and 2876 for NRCWE-049). The main biological process (Gene ontology) common a specific for MWCNT studied were presented (DAVID analysis)

In order to study gene expression variation after exposure to the functionalized MWCNT and to understand toxicity mechanism induced by these CNT, a transcriptomic study was performed. The IC_50_ determined according to WST-1 assay were utilized to determine the dose to be employed in the transcriptomics study, namely at ¼ IC_50_, a subtoxic doses that allowed to study the primary responses directly related to the exposure to these CNT, not the secondary ones and in order to identify initiating key events leading to their toxicity. Transcriptomic analysis showed significant genes expression modifications following exposure to each of these 3 nanoparticles. After Gene Spring normalization, we have 6049 differentially expressed genes (DEG), at a p value < 0.001 and FC cut-off level of 1.5, for the amino functionalized CNT NRCWE-049, 3388 DEG for the carboxyl CNT NRCWE-042 and 245 for the nonfunctionalized CNT NM403. The functionalized CNT NRCWE-049 and NRCWE-042 provoked a higher number of deregulated genes compared to the nonfunctionalized NM403: respectively 6049 > 3388 > 245 genes (Fig. 3).

The DAVID database was also used to analyze gene expression data that does not give information on pathway relationship analyzed using IPA software but instead reveals significant clusters of genes. From the DAVID analysis, among 48 common genes (Fig. 3, diagram 2, FC ≥ 1.5) the main cluster was related to positive regulation of Rho protein signal transduction. These proteins are involved in cytoskeletal dynamics, so we can link this common response to the tubular shape of CNT that may lead to cytoskeletal damage one they are internalized and thus to intracellular dynamics perturbation. Genes which were differentially expressed between the three MWCNT were related to (i) oxidative stress response or drug transmembrane transport for NM403, (ii) positive regulation of cytokine production for NRCWE-049 and (iii) translation for NRCWE-042 (FC ≥ 1.5).

No common deregulated pathway was identified for the three MWCNT based on Ingenuity Pathway Analysis (IPA) software but there were four pathways among the top canonical ones (FC ≥ 1.5), in common between functionalized CNT NRCWE-042 and NRCWE-049 included namely mitochondrial dysfunctions, eukaryotic initiation factor-2 (EIF2) signaling, Sirtuin signaling and oxidative phosphorylation (Table 2). These pathways are related mainly to mitochondrial damage and proteins synthesis perturbation since EIF2 which is an essential factor to an efficient translation proceeding, was dysregulated. For NRCWE-049, inflammasome activation was identified among the top canonical pathways and was presented in Fig. 5. Pathways presented in this table and discussed in this study were the top enriched group of genes.

**Table 2:** Gene expression analysis by IPA software at FC ≥ 1.5 and p value < 0.001 showing the top canonical pathways for each of MWCNT studied

Common pathways	Differentially regulated pathways		
No common pathway between 3 MWCNT	NM403	NRCWE-042	NRCWE-049
	Role of Brca1 in DNA damage Atm signaling Vitamin-C Transport Cell cycle: G2/M DNA damage checkpoint regulation NrF2 mediated oxidative stress response	Common	
		Mitochondrial dysfunction EIF2 signaling Sirtuin signaling Oxidative phosphorylation	
		Different	
		Regulation of eIF4 and p70S6K signaling	mTOR signaling

Transcriptomic study was performed using NR8383 cells exposed to ¼ IC_50_ of each MWCNT for 4 h (N, number of biological replicates = 4). The table shows common and differentially regulated pathways between three CNT

**Fig. 4:**
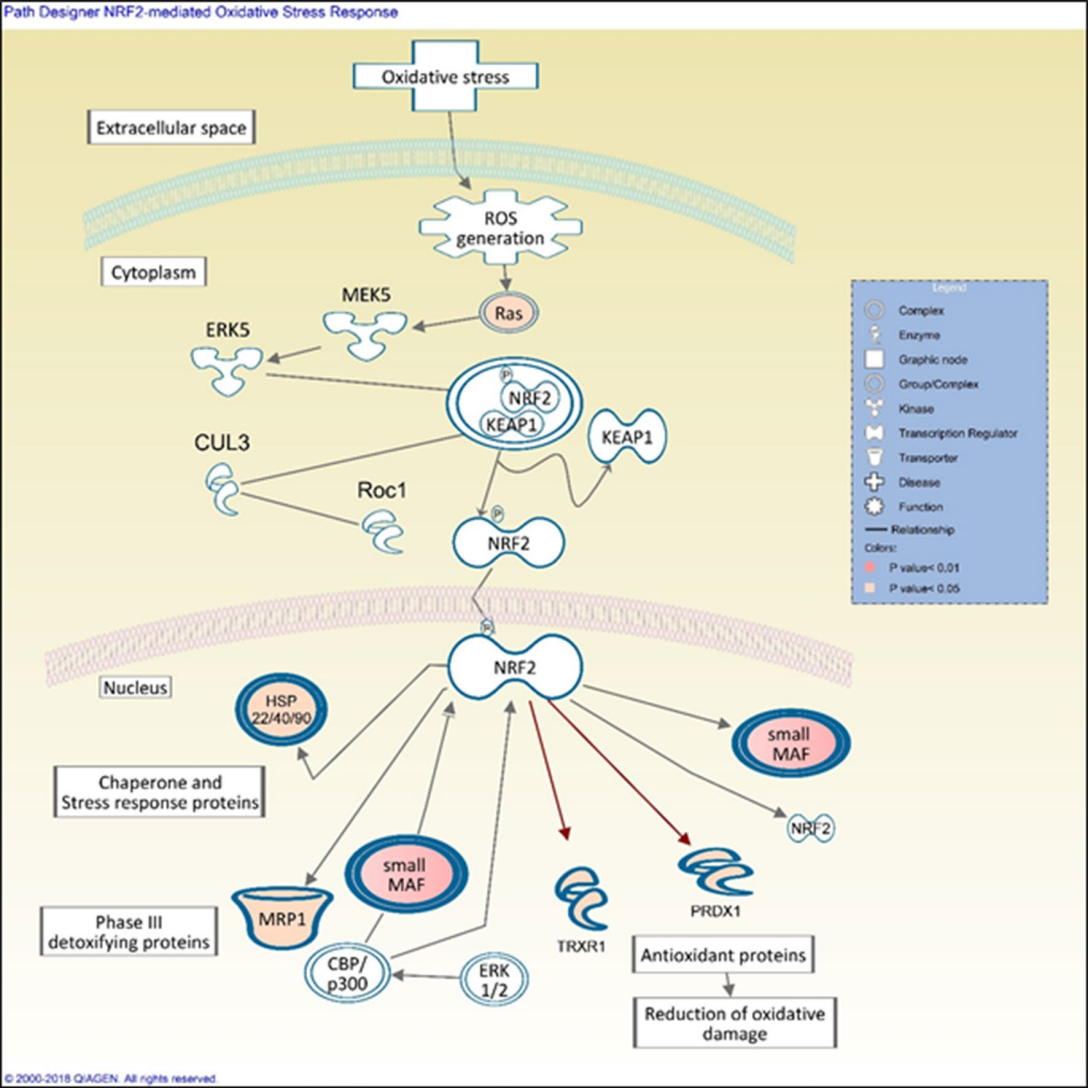
Gene expression analysis by IPA software (FC ≥ 1.5 and p value < 0.001) showing nuclear factor erythroid 2-related factor 2 (NRF2) oxidative stress response path design activated in NR8383 cells after exposure to ¼ IC_50_ of NM403 for 4 h. Only upregulated genes implicated in this pathway are shown. Double bold line means that we have a complex

**Fig. 5:**
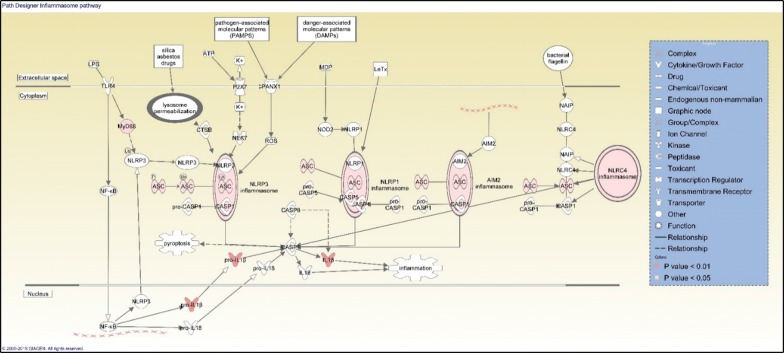
Inflammasome path as represented by IPA software (FC ≥ 1.5 and p value < 0.001) showing upregulated genes after NR8383 cells exposure to ¼ IC_50_ of NRCWE-049 for 4 h

For NM403, the nuclear factor erythroid 2-related factor 2 (NRF2) oxidative stress response pathway (Fig. 4) and the breast cancer gene 1 (*Brca1*) involved in DNA damage response pathway was inhibited (Appendix). The activation of NRF2 pathway correlates with David analysis findings saying that the main significant cluster was related to oxidative stress response in NM403 case. Figure 4 represents DEG involved in NRF2 pathway and showed that oxidative stress response was passed through RAS signaling. Figure 5 showed that NRCWE-049 the amino functionalized CNT induced an activation of inflammasome subunits NLRP1 NLRP3 NLRC4 and AIM2.

### Proteomic study

#### Whole proteome analysis

**Fig. 6:**
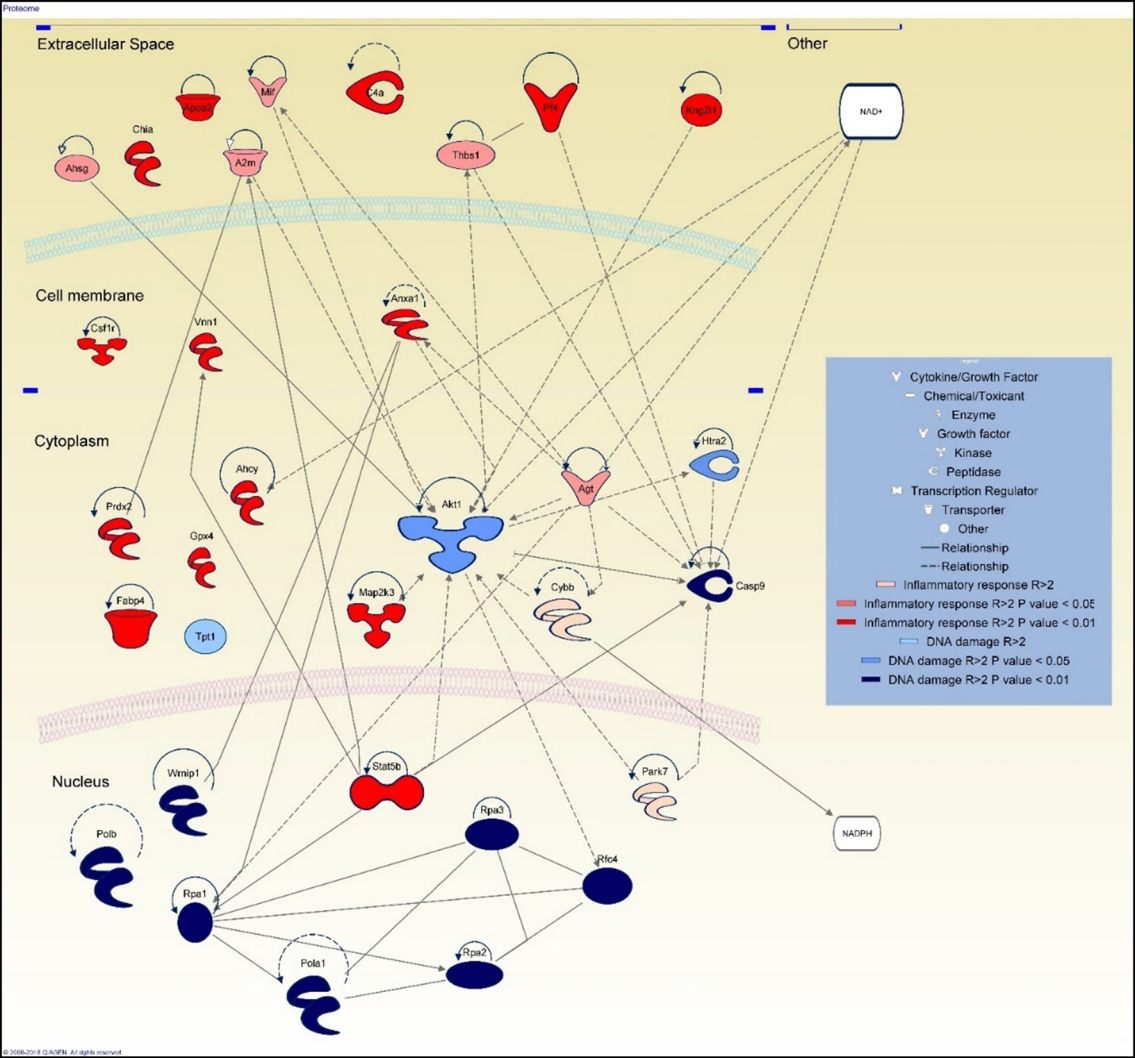
Supernatant and whole cell lysate proteins analyzed by IPA software. The stringency was fixed to R > 2, show proteins which were implicated in DNA damage and inflammatory response. Proteomic study was performed using NR8383 cells exposed to ¼ IC_50_ of NM403 for 24 h (N = 4)

In order to gain a better understanding of the effects of CNT exposure on the macrophage cells the cytotoxicity and transcriptomics analysis was supplemented by a proteomic study. The most significant DEG that are been identified according to transcriptomic data were also expressed at the protein level according to proteomics ones. Only those genes that are expressed also at the protein level were took onto consideration as relevant and significant to conclude about NM403 toxicity potential. NM403 whole proteome and secretome study showed an increase in protein synthesis related to inflammatory response, acute inflammation, chronic inflammation and DNA damage which was also conclude according to transcriptomic analysis (Fig. 6).

**Fig. 7:**
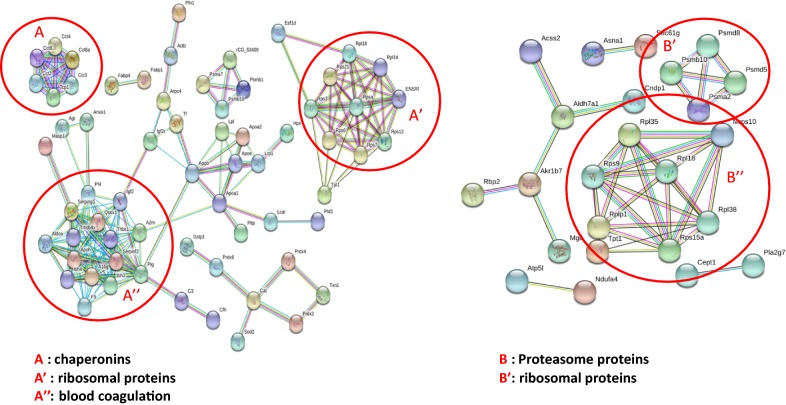
Supernatant and whole cell lysate proteins analysed by String database performed at high confidence (R > 2) show main protein clusters: chaperonins, ribosomal protein, proteasome and blood coagulation proteins. Proteomic study was performed using NR8383 cells exposed to ¼ IC_50_ of NM403 for 24 h (N = 4)

Among the list of supernatant proteins which are expressed after exposure to NM403, Vimentin was the most highly produced protein detected in the NR8383 cells supernatant. String analysis was performed at a high confidence setting using only the proteins identified in the supernatant which were shown to be increased twofold greater in CNT exposed macrophages in comparison to the control cells (R > 2). This analysis indicated the presence of several different subgroups within the protein network including chaperonins, i.e. CCT4, CCT8, CCT3; ribosomal proteins, i.e. RPS3, RPS12, RPL18; and blood coagulation proteins (Fig. 7). Ribosomal proteins were also observed to be increased in the whole cell proteomics analysis (R > 2) in addition to proteasome proteins, i.e. PSMA2, PSMD8, PSMD5 (Fig. 7).

### Cytokine array

**Fig. 8:**
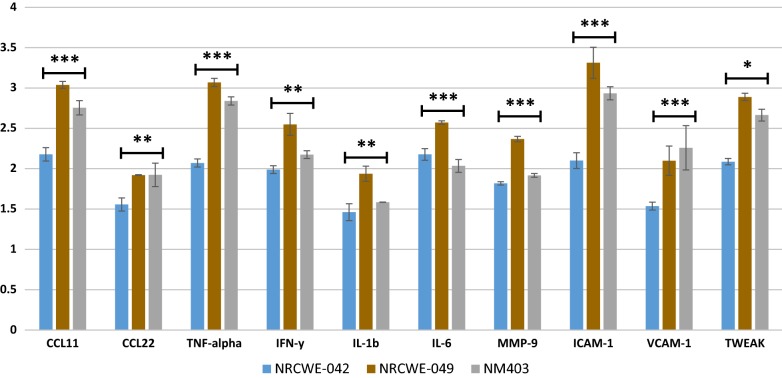
Densitometric analysis relative to control of a group of proinflammatory cytokines (*CCL11*, *CCL22*, *TNF*-*alpha*, *IFNy*, *IL*-*1b*, *IL*-*6*, *MMP*-*9*, *ICAM*-*1* and *VCAM*-*1*) from supernatant of cells exposed to ¼ IC50 NRCWE-042, NRCWE-049 or NM403. TWEAK, a cytokine that could play a role in autophagy induction has been added. Densitometric analysis are compared to the control. Means are represented ± SE using ANOVA. *p < 0.10, **p < 0.05, ***p < 0.01

Inflammatory reaction was the main response induced by all CNT according to both transcriptomic and proteomic data. In order to prove the inflammatory potential of each CNT and to compare between them, thus deducing the role of functionalization in inflammatory reaction induction and potency, we performed a cytokine array. An analysis of 79 cytokine expression shows a global augmentation in the expression of different cytokines already expressed in our control. This result is observed after NR8383 exposure to each MWCNT. A group of the most produced cytokines was involved in inflammatory response and has been presented in Fig. 8 of which: CCL11, CCL22, IL-1β, IL-6, IFNγ and TNFα. By the way MMP-9, ICAM-1 and VCAM-1 were also overexpressed. Regarding results specific to each MWCNT used, a lower cytokine secretion level is observed for cells exposed to NRCWE-042 compared to NRCWE-049 or NM403. Cells exposed to NRCWE-049 gives highest cytokine secretion level for most of the studied cytokines.

## Discussion

Each MWCNT studied employed in this study had similar Z-averages (212.7 ± 65.6) in DMEM medium supplemented with 2% FBS. It is well known that functionalized MWCNT agglomerate due to interactions with proteins [40], therefore both NRCWE-042 and NRCWE-049 required higher sonication amplitude to produce an adequately dispersed solutions (Table 1). The study shows that the carboxyl-MWCNT NRCWE-042 was less toxic than the pristine one NM403 and the amino-MWCNT one NRCWE-049 (Fig. 2). These findings are in agreement with by Seyed Yazdan Madani et al. [41] demonstrated that HT29 cells exposed to carboxylic CNT expresssed higher viability than those exposed to pristine CNT. This may be due to their interaction with acidic proteins, forming important aggregates which mitigate their toxicity [40]. Also, it can be due to a smaller diameter of pristine MWCNT NM403 and amino-MWCNT NRCWE-049 compared to the one of carboxyl-MWCNT NRCWE-042 [42]. For amino functionalization, studies have demonstrated no significant variation in toxicity in comparison to the pristine CNT form [21]. Also, a study by Loos et al. [43] demonstrated enhanced CNT associated toxicity when functionalized with an amino group in comparison to a carboxyl group. Therefore, it can be concluded that functionalization nature performed will have a significant impact on downstream toxicity. However, it is also important to note that the observed toxicity appears to be linked to the chosen in vitro model, as a work reported low levels of toxicity in PC12 cells in response to amino-MWCNT [22], in contrast to the high toxicity observed in three leukemia cell lines, THP-1, U-937 and HL-60, where the amino-CNT prevented cell proliferation [43]. As regards the pristine NM403, the cytotoxicity observed may be a result of short length compared to bothNRCWE-042 and NRCWE-049 ones [44].

### Common toxicity mechanism of MWCNT

Functionalized MWCNT gene expression analysis highlighted an overexpression of genes related to inflammatory processes, including: *Cxcl2*, *Ccrl2*, *Il6r*, *Il10ra*, *Il17re* and *Ilf2* for NRCWE-049 and *Ccl2* and *Ccl4* for *NM403*, and *Irak2*, *Il7r*, *Il1b*, *Il1a*, *Socs3* and *Ifit3* for NRCWE-042. For NM403, only proteomics analysis identified inflammatory proteins (Fig. 6), including *Anxa1*, *Apoa2*, *c4A*, *Chia*, *Gpx4*, *Kng2l1*, *MAP2K3*, *Prdx2*, *Stat5B* and Vnn1 that were overexpressed (p < 0.01) and A2m, Agt, Ahsg, Mif and Thbs1 (> twofold increase, p < 0.05). These findings are consistent with a previous study of our team that showed an overexpression of *TNFA* and *Il1b* in response to exposure to pristine MWCNT NM403 [30]. Otherwise, according to results obtained by cytokine assay, these cytokines were also secreted by rat alveolar macrophages NR8383 exposed to ¼ IC_50_ of each MWCNT. Expression level augmentation has notably been observed for proinflammatory cytokines such as CCL11 and CCL22 (Fig. 8) which are different chemokines needed to recruit cells populations from immune system [45]. IFNγ and TNFα also showed an increase in expression level, which are well known proinflammatory cytokines. However, a lower increase of proinflammatory cytokine secretion was observed when cells were exposed to NRCWE-042 comparing to NRCWE-049 or NM403. This result is consistent with previous experiments underlining a lower toxicity of –COOH functionalized MWCNT. Therefore this study provides evidence that both functionalized and non-functionalized MWCNT induce inflammatory response, in agreement with a study by Pescatori et al. [46]. In addition to inflammatory processes, exposure of NR8383 rat alveolar macrophage cells to every MWCNT results in the deregulation of Rho proteins coding genes (Fig. 3) and tubulin genes according to gene expression data (*Tuba1a*, *Tuba4a*, *Tubb6* and *Tubb2b*). Proteomic analysis confirmed the transcriptomics finding for pristine MWCNT NM403, identifying increased protein expression of proteins involved in Tubulin proteins synthesis, i.e. Tubb4b, Tubb5a, Tub1ab, as well as chaperonin proteins which are responsible for proper tubulin actin folding [47], i.e. Cct2, Cct3, Cct4, Cct8, showing that MWCNT may induce cytoskeleton disorganization (Fig. 7). Interestingly, the actin cytoskeleton pathway was shown to be the most deregulated pathway in response to the NRCWE-049 according to gene expression analysis. It can be proposed from these findings that the needle-like shape of NRCWE-049 induces cytoskeleton effect as it has been shown that cationic MWCNT’s positive charge promote their internalization, thus eliciting more damage to cytoskeleton than the pristine or anionic MWCNT [48].

### Common mechanism of functionalized MWCNT (ER) stress

Exposure to pristine MWCNT NM403 was shown to result in the dysregulation of ribosomal genes and lead to the unprogrammed synthesis of ribosomal proteins. We have a disruption of ribosomal proteins, proteasome proteins and chaperonin proteins which are involved in the correct folding of proteins (Fig. 7). Consequently, NM403 induced endoplasmic reticulum (ER) stress by disrupting proteins homeostasis. The perturbation of these proteins can be linked to inflammatory response induction. Chaperonin can stimulate the production of pro-inflammatory cytokines and other proteins involved in immunity and inflammation [49]. Also, the perturbation of ribosomal and proteasome proteins leading to proteins accumulation could trigger some pathways such interferon pathway thus promoting inflammatory reaction [50]. However, an overexpression of severed genes including *Grb2*, *Igf2bp2*, *Vegfb*, *Gfer* and Pdgfa indicated a dysregulation of mTOR pathway was observed only with functionalized MWCNT, NRCWE-042 and NRCWE-049 [35]. Indeed these MWCNT induced ER stress as a result of these alterations in the mTOR signaling pathway. In addition, an overexpression of *Lamtor4* and ribosomal genes, including *Rpl39l*, *Rps27* and *Rps14*, following MWCNT exposure occurred. NRCWE-042 and NRCWE-049 overexpressed *Lamtor4*, a part of the Ragulator complex, that is involved in mTORC1 activation [51, 52] that in turn increases translation of mRNA associated to ribosomes [53]. Therefore this study proposes that all MWCNT studied induce ER stress, however, their functionalisation provoked mTOR dependent ER stress. This finding is in agreement with a previous study published Lunova et al. [54].

### Functionalization-dependent mechanisms

#### Toxicity mechanism of non-functionalized MWCNT, NM403

Exposure of NR8383 cells to pristine MWCNT NM403 only activatedNRF2 pathway according to transcriptomic analysis (Table 1). NRF2 plays a role in maintaining the balance between oxidative stress and antioxidant defense activated by genes such as *Abcc1*, *Dnajc7*, *Maff*, *Mafg*, *Prdx1* and *Txnrd1*, of which expression was found to be upregulated. Figure 4 shows the NRF2 pathway and highlights all those genes which were overexpressed following treatment. In addition, the Vitamin C transport pathway was shown to be dysregulated, in conjunction with the overexpression of the *Slc23a2* gene, both of which have been implicated in antioxidant response. Previously Vales et al. showed increased ROS production after exposure of BEAS-2B cells to the same nanomaterial [44]. Taken together, we conclude that pristine MWCNT NM403 induced ROS generation, which subsequently leads to the activation of the NRF2 response pathway. It should be mentioned that ROS generation can result from the percentage of impurities present in this CNT (Al, Mg, Na, Mn and Co) which is the highest between studied MWCNT as it has been shown previously [55].

Pristine MWCNT NM403 exposure was also demonstrated to have a significant impact on DNA damage response signaling. Several pathways related to different factors of DNA damage response were shown to be dysregulated in response to NM403 exposure; including checkpoint kinases: CHK1 and CHK2; cell cycle: G1/S checkpoint regulation; cell cycle: G2/M DNA damage; Ataxia telangiectasia-mutated protein kinase: ATM signaling, and DNA damage proteins e.g. Brca1 (Table 2). Additionally, *Rad51*, which is known to be involved in DNA repair [56], was significantly overexpressed (FC = 2.6). Furthermore, Brca1, a tumor suppressor, which is crucial for cell cycle checkpoints, DNA damage sensitivity and efficient ATM signaling [57, 58], which ensure high-fidelity HR repair [59], was inhibited (Appendix). According to these results, NM403 seems to be an inducer of DNA damage which can be connected to ROS generation induced by pristine MWCNT NM403 [60, 61].

Consequently, impairment of DNA damage repair mechanisms may lead to DNA damage accumulation, thus explaining the increased NR8383 cell death detected by LDH assay following exposure to pristine MWCNT NM403 [62]. Previous studies have indicated that DNA damage [63] and cell death [64] contribute to inflammatory response. Additionally, our analysis indicated that interferons, which are known to be involved in DNA damage [65] and the induction of inflammatory response [66], were dysregulated following exposure to the pristine MWCNT NM403. Therefore, it can be concluded that pristine MWCNT NM403 contributes to the onset of inflammation through the induction of DNA damage and cell death.

#### Toxicity mechanism of cationic MWCNT, NRCWE-049

Transcription factor EB (TFEB), protein coding gene which acts as a positive regulator of autophagy by promoting expression of genes involved in autophagy, was shown to be overexpressed only in response to the cationic MWCNT, NRCWE-049. This finding is supported by the activation of TFEB in the presence of cations [67]. Amino-MWCNT NRCWE-049 also increased expression of autophagy-related genes: *Atg9a*, *Atg2a*, *Atg16l1*, *Atg101* and *Atg4d*, that can be linked to the activation of TFEB. Furthermore, NRCWE-049 was also shown to result in dysregulation of the mTOR pathway, which has recently been shown to promote autophagy [54]. Therefore, the findings from this study support an induction of autophagy by NRCWE-049. Another interesting result during cytokine proteomic analysis was about TWEAK expression levels. The later acting with others such as IL-1β, IL-6, IFNγ and TNFα could play a role in autophagy induction [68]. TWEAK expression level was found the highest in supernatants of cells exposed to amino-MWCNT NRCWE-049. This is a complementary fact explaining autophagy induction by amnio-MWCNT NRCWE-049. MayNRCWE-049, in a similar manner to other cationic CNT, induces the permeabilization of lysosomes through proton leaching, which is known as “Sponge Effect” [54], thereby preventing the anchoring of the mTORC1 complex to the lysosomal membrane. This would subsequently result in the inhibition of mTOR and the induction of autophagy, as proposed by Liu et al. [69], and could explain the mitochondrial dysfunctions observed in response to these MWCNT (p value of mitochondrial dysfunction pathway was the highest with NRCWE-049, p = 1.17E−25). Furthermore, NRCWE-049 exposure contributed to inflammasome activation (Fig. 5) which may result from lysosome destabilization and cause the release of these inflammatory mediators, including *Cxcl2*, *Ccrl2*, *Cklf*, *Crlf2*, *Il6r*, *Il10ra*, *Il17re*, *Il3ra*, *Ilf2* and *Nfil3*, an occurrence which has previously been observed in alveolar macrophages following exposure to silica nanoparticles [70]. Therefore, our study proposes that exposure to cationic MWCNT induces lysosomal destabilization, which functions as a molecular initiating event triggering ROS formation and mitochondrial dysfunction, leading to inflammasome activation and cell death via autophagy. Thus, lysosomal stress can be considered a marker of toxicity for cationic MWCNT. Otherwise, this lysosomal stress can be charge independent, and it could be a result of amino group presence as it was the case with chloroquine or Eudragit nanoparticles inducing lysosomal stress either in primary rat hepatocytes or in NR8383 cells [71].

#### Toxicity mechanism of anionic MWCNT, NRCWE-042

Our transcriptomic analysis highlighted dysregulation of the mTOR and EIF4/p70S6K signaling pathways in response to NRCWE-042 exposure (Table 2). Moreover, the data highlighted an upregulation of ribosomal protein S6 genes, including *Rps6ka1*, *Rps6ka4*, *Mrps2* and *Mrps6*, as well as dysregulation of genes related to translation, such as *Eef1g*, *Eif1b*, *Eif6*, *Eif2s2*, *Eif1a*, *Eif4ebp1*, *Eif1*, *Eif3m*, *Eif3k*, *Tpt1* and *Eif3*. A study by Lunova et al. [54] showed that anionic CNT do not induce lysosomal stress. Indeed following carboxyl MWCNT NRCWE-042 exposure we do evidence any lysosomal stress but an increase of *Lamtor* expression, thereby activating mTOR signaling which in turn controls the translational machinery, activating p70 S6 kinase protein (p70S6k) and inhibiting the eIF-4E inhibitor, the 4E-BP1 molecule. This results in the activation of the 40S ribosomal protein S6, which is known to contribute to the translation of 5′-TOP mRNAs encoding ribosomal proteins and components of the translational apparatus [72, 73]. As a result of the increased translational machinery activity, increased interferon signaling, through Ifngr2 (FC = 2.3) and Ifit3 (FC = 69.3), was identified, in order to alter mRNA translation through mTOR inhibition. As previously mentioned, interferon signaling is linked to inflammatory response. Further evidence for the role of interferon signaling in inflammation is provided by the over-expression of the *Tlr2* gene (FC = 6.8) observed in response to NRCWE-042 exposure, which is known to be implicated with interferon gamma signaling in inflammatory response induction [74–77]. Based on the findings of this study, NRCWE-042 triggers both interferon and Tlr2 signaling to induce inflammatory response in NR8383 alveolar macrophage cells.

According to our study, the functionalization of CNT with amino groups increases their toxicity, which was not well known, to our knowledge. They have the highest inflammatory potential, they produce an activation of inflammasome, a key event which may lead to chronic inflammation as reported for silica nanoparticles [78]. Inflammasome activation is associated to some pathologies such as lung cancer, silicosis and mesothelioma these CNT may be involved in such pathologies. Proving that functionalization of CNT with amino groups influence their toxicity potential, in contrary to some literature results, may prevent its use in nanomedicine applications. Our study would contribute to the design of more secure CNT.

## Conclusion

According to the presented results of this study, all three MWCNT lead to inflammatory response and ER stress which could potentially function as a biomarker of toxicity for all MWCNT. In contrast lysosomal destabilization was shown to be a potential biomarker for the cationic MWCNT, NRCWE-049. Interestingly, the type of functionalization alters the response of macrophages to MWCNT, inducing mTOR dependent signaling pathways which can result in either autophagy or enhanced translation depending on the MWCNT charge (Fig. 8). This study by combining transcriptomic and proteomic screenings provide better understanding of functionalized CNT toxicity mechanisms in rat alveolar macrophages, which can be also concluding for humans who are repeatedly exposed to CNT, especially workers in factories. It is also important to move toward safer CNT design.

## Figures and Tables

**Fig. 9:**
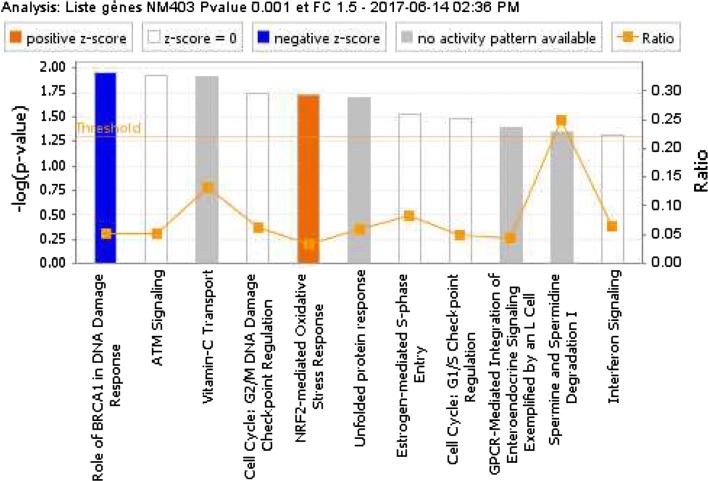
Gene expression analysis by IPA software at FC ≥ 1.5 and p value < 0.001 showing canonical pathways of NM403. Transcriptomic study was performed using NR8383 cells exposed to ¼IC_50_ of NM403 for 4 h. N, number of biological replicates, was equal to four

## Data Availability

All data generated or analysed during this study are included in this published article. According to European funding rules, all data will be made available to public within 2 years after the end of the Smartnanotox project.

## Appendix

See Fig. 9.
